# Integration of Descending Command Systems for the Generation of Context-Specific Locomotor Behaviors

**DOI:** 10.3389/fnins.2017.00581

**Published:** 2017-10-18

**Authors:** Linda H. Kim, Sandeep Sharma, Simon A. Sharples, Kyle A. Mayr, Charlie H. T. Kwok, Patrick J. Whelan

**Affiliations:** ^1^Hotchkiss Brain Institute, University of Calgary, Calgary, AB, Canada; ^2^Department of Neuroscience, University of Calgary, Calgary, AB, Canada; ^3^Department of Comparative Biology and Experimental Medicine, University of Calgary, Calgary, AB, Canada

**Keywords:** locomotor behavior, supraspinal, descending, goal-directed, approach, aversion

## Abstract

Over the past decade there has been a renaissance in our understanding of spinal cord circuits; new technologies are beginning to provide key insights into descending circuits which project onto spinal cord central pattern generators. By integrating work from both the locomotor and animal behavioral fields, we can now examine context-specific control of locomotion, with an emphasis on descending modulation arising from various regions of the brainstem. Here we examine approach and avoidance behaviors and the circuits that lead to the production and arrest of locomotion.

## Introduction

Animals produce a wide array of locomotor behaviors in response to internal and external cues. Normally, animals survey the environment in search of appropriate olfaction, audition, visual, or tactile sensory inputs. Internally motivated cues may be due to appetitive drive such as food and reproduction, and other physiological needs like safety, shelter, or adaptation to a new environment. These cues inform ongoing movement sequences by converging onto locomotor control centers in the brainstem and spinal cord, thus facilitating the generation of context-appropriate locomotor behaviors.

In the first part of this review, we focus on key supraspinal regions for locomotor control, with emphasis placed on how technological advances are beginning to reveal cell types and the underlying functional connectome. In the second part of this review, we will explore the afferent projections to these locomotor regions and discuss how internal and external triggers can drive appetitive (approach) or aversive (avoidance) locomotor responses.

## Descending command systems for locomotion

**Figure 1:**
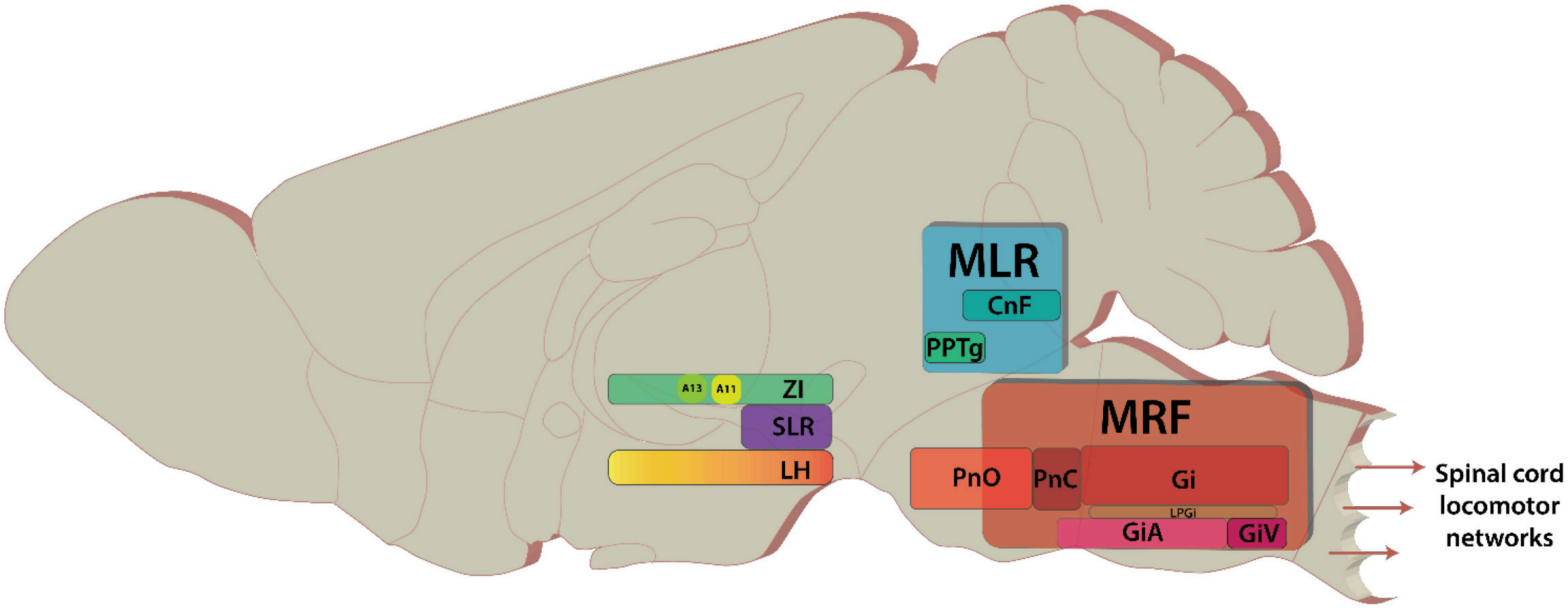
Key areas of the brain discussed in the review. The brain areas are included here have diverse functions in addition to modulating locomotion. Sagittal view of representative locations of locomotor centers.

Over the past 75 years, studies on the descending control of locomotion have been directed toward three regions (Whelan, 1996; Jordan et al., 2008); the Subthalamic Locomotor Region (SLR), the Mesencephalic Locomotor Region (MLR: Figure 1), and the Medullary Reticular Formation (MRF: Figure 1). These regions were initially identified based on their ability to elicit various forms of locomotor behaviors in response to direct electrical stimulation of these regions. The term “locomotor region” was used since electrical stimulation cannot be confined to anatomically-defined nuclei. These regions are conserved across vertebrate species studied (reviews Rossignol et al., 2006; Fetcho et al., 2008; Grillner et al., 2008; Jordan et al., 2008; Ryczko and Dubuc, 2013) with initial experiments being performed in cats (Shik et al., 1969—English translation of their 1966 publication; reviews Armstrong, 1986; Whelan, 1996), later in rats (Mel'nikova, 1975, 1977 as cited by Ross and Sinnamon, 1984; Sinnamon et al., 1984; Skinner and Garcia-Rill, 1984), and recently in mice (Bouvier et al., 2015; Roseberry et al., 2016).

Investigations into the underlying cellular and system mechanisms for the generation of locomotor behaviors begun in tandem with the identification of locomotor regions. Earlier work was performed in relatively simple organisms (Gillette et al., 1978; Olson and Krasne, 1981; Edwards et al., 1999; Esch and Kristan, 2002) and primitive vertebrates (Grillner et al., 2008). Work on the leech, lamprey and xenopus have provided fascinating similarities between swim behaviors and circuit organization of organisms separated by up to 560 million years of evolution (Mullins et al., 2011). Recent advances in molecular biology and genetic tools have enabled the functional connectivity of locomotor circuit elements in mammalian systems to be explored (Bouvier et al., 2015; Roseberry et al., 2016).

## The medullary reticular formation: a centre for locomotor stop and go

The MRF contains groups of diffusely located nuclei that form an important integration center for the control of locomotion, with descending projections onto interneurons and motoneurons of the cervical and lumbar spinal cord (Grillner et al., 1968; Peterson et al., 1979; Bouvier et al., 2015). Shik et al. (1969) were the first to show the existence of a pathway from the MLR to the MRF. Although, activity in the MRF is known to correspond to locomotor activity, recordings from the MRF in freely walking cats showed that activity patterns were complex, as only some units corresponded to the rhythmic electromyographic activity of muscles while others did not (Drew et al., 1986; Perreault et al., 1993). Both electrical and direct drug-based stimulation of the MRF can elicit locomotion (Garcia-Rill and Skinner, 1987a,b; Noga et al., 1988; Jordan, 1998). Similarly, rhythmic patterns or locomotor-like rhythmicity in the isolated *in vitro* brainstem-spinal cord preparation can be evoked (Liu and Jordan, 2005; Hägglund et al., 2010; Kiehn, 2016). Additionally, stimulation of ventrolateral funiculi, containing the reticulospinal projections, can produce bouts of rhythmic motor activity (Magnuson et al., 1995). Acute lesions of this tract eliminate locomotion elicited by stimulation of the MLR in decerebrate cats (Steeves and Jordan, 1980) and result in changes of gait in freely moving animals (Brustein and Rossignol, 1998).

Classically, the MRF has been subdivided into four key nuclei distributed across the medulla and the pons and is also known as ponto-medullary reticular formation (review Drew et al., 2004). In rodents, these regions correspond with pontine reticular nucleus oral (PnO) and caudal (PnC), gigantocellular reticular nucleus (Gi), and magnocellular nucleus of medulla which encompasses lateral paragigantocellular nucleus (LPGi), gigantocellular reticular nucleus alpha (GiA), and ventral section (GiV), respectively (Paxinos and Franklin, 2008; Esposito et al., 2014). Recent work has focused on the Gi, LPGi, GiA, and GiV. These nuclei form the reticulospinal pathway, and contain cells that descend ipsilaterally via the ventrolateral and ventromedial funiculi (Petras, 1967; Peterson et al., 1979). Contralateral projections also exist (Jankowska et al., 2003; Krutki et al., 2003; Szokol et al., 2011). Recent work using genetic and intersectional viral tracing has revealed that reticulospinal pathways are more diverse than previously thought, and cells can be organized into clusters based on their projections either in the cervical or lumbar spinal cord (Esposito et al., 2014). The ventral part of the MRF (MdV) was highlighted as a region with functional connectivity to forelimb motor neurons suggesting that MRF regions may be organized somatotopically. Interestingly in cat MRF cells were observed to project over long distances to multiple segments (Matsuyama et al., 1999). These differences may represent the different demands of grasping for rodents compared to cats (Whishaw et al., 2008). However, projections to multiple segments have also been observed in the monkey (Kneisley et al., 1978; Coulter et al., 1979).

Most descending MRF cells are glutamatergic and were traditionally thought to form the excitatory command signal for locomotion. Photostimulation of vesicular glutamate 2 (Vglut2)-expressing cells in the brainstem with channelrhodopsin 2 (ChR2) can elicit spinal rhythmicity *in vitro* (Hägglund et al., 2010). Subsequent investigations explored a subpopulation of glutamatergic cells in the brainstem that can be identified by the Chx10 and Lhx3 transcription factors. These cells express c-fos (a marker of neuronal activation) following bouts of locomotor activity, receive input from the MLR, and project to the cervical spinal cord (Bretzner and Brownstone, 2013). Manipulation of activity of these cells *in vivo* did not alter locomotor behavior in mice (Bretzner and Brownstone, 2013), however, activation of these cells in zebrafish is sufficient to drive locomotor activity (Kimura et al., 2013). Surprisingly, recent work on the Chx10 population in mice found that activation of this cell-type at the junction between the rostral medulla (rostral Gi) and caudal pons (PnC) disrupted spinally-generated rhythmicity *in vitro*, and caused the animal to stop when activated *in vivo* (Bouvier et al., 2015). Recruitment of spinally-located premotor inhibitory interneurons by the Chx10 MRF neurons was implicated (Bouvier et al., 2015). Conversely, stop commands in fish (Wannier et al., 1995) and tadpoles (Boothby and Roberts, 1992; Perrins et al., 2002; Li et al., 2003) are reported to be mediated by descending inhibitory cells of the MRF, which project mono-synaptically to motoneurons and are triggered by sensory afferents in the head. This poses an interesting possibility for a parallel stopping mechanism in mammals that remains to be discovered (review Klemm, 2001). Indeed, a GABAergic/glycinergic projection from the MRF to the spinal cord exists in rodents but its function in locomotor control remains unknown (Holstege, 1991).

In addition to the glutamatergic and GABAergic/glycinergic descending pathways within the brainstem are several monoaminergic neuromodulatory pathways that can modulate locomotor activity. The major descending brainstem modulatory pathways are the serotonergic raphespinal pathway and the noradrenergic coeruleospinal pathways. These systems have been reviewed elsewhere (Jordan et al., 2008). Recent work suggests that monoamines (5-HT and noradrenaline) increase in concentration many seconds before locomotion and decrease gradually to baseline once locomotion is terminated. These long timeframes suggest that monoamines are not involved in moment-to-moment modulation of spinal cord circuits and may possibly be released extrasynaptically (Noga B. R. et al., 2017).

## The mesencephalic locomotor region: an integrative hub for locomotor speed and gait

The MLR is located on the mesopontine border and comprises of the cuneiform (CnF) and the pedunculopontine nuclei (PPN) (Figure 1). In basal vertebrates, the MLR comprises the laterodorsal tegmental nucleus and the PPN. In mammals, it comprises the PPN, but also the CnF (Ryczko and Dubuc, 2013, 2017). It can be described as a classical region for locomotor control. Electrical stimulation of the mesopontine border with increasing intensity led to a serial progression from walking to running to galloping (Shik et al., 1969). Initially it was thought that the MLR projects serially to the MRF and acts as a “volume-control” for locomotion, as ablation of the MRF abolished MLR-evoked responses in the spinal cord (Noga et al., 2003). Since the original finding the location of the nuclei comprising the MLR and its projections have been steadily refined.

The PPN, referred to as the pedunculopontine tegmental nucleus (PPTg) in rodents (Paxinos and Franklin, 2008) and sometimes the nucleus tegmenti pedulculopontinus in humans (Schaltenbrand and Wahren, 1977), is located in the ventrolateral portion of the MLR (Olszewski and Baxter, 1954). It is composed of cells with heterogeneous neurotransmitter phenotypes including but not limited to: GABA, glutamate, acetylcholine, and calcium-binding proteins and neuropeptides (Clements and Grant, 1990; Lavoie and Parent, 1994; Fortin and Parent, 1999; Vincent, 2000; Mena-Segovia et al., 2008, 2009). There is no doubt that the use of different terms has led to confusion in the field. In this review we will refer to the PPTg when discussing rodent relevant papers and PPN when referring to cat, monkey, or human work. In terms of projections, the PPN and CnF connect to sensorimotor, associative, and limbic areas of the basal ganglia and the thalamus in monkeys and humans (Sébille et al., 2017). Analysis of these projections suggests that the PPN may integrate sensorimotor, cognitive, and emotional information. The anterior part of the PPN may be related to motor control in the monkey. In contrast, the CnF connectome is more restricted involving predominantly limbic brain regions (Sébille et al., 2017). Some reports have identified the PPTg as an effective site for evoking locomotion, and direct efferent projections to the lumbar spinal cord have been reported (Skinner et al., 1990). Of the two regions, stimulation of the CnF appears to be more robust in driving locomotion in the cat (Shik and Orlovsky, 1976—original article in Russian: Sirota and Shik, 1973). The CnF lies dorsal to PPTg and borders the inferior colliculi ventrally (Allen Brain Atlas: https://tinyurl.com/k8g98tl). Like the PPTg, the CnF is composed of heterogenous cell types including GABAergic (Ford et al., 1995), glutamatergic (Heise and Mitrofanis, 2006), peptidergic (Sar et al., 1978; Beitz, 1982a,b) cells with some cholinergic neurons (Ford et al., 1995).

Recent work examined MLR cell types for their roles in locomotor behaviors (Roseberry et al., 2016; Kroeger et al., 2017; Mena-Segovia and Bolam, 2017). Activity of the glutamatergic MLR cells correlate with spontaneous locomotor episodes and their activation is sufficient to produce locomotor bouts. Conversely, activity of the GABAergic population is associated with stationary states and their activation stops locomotion, partly through suppression of the local MLR glutamatergic population (Roseberry et al., 2016). This suggests that GABAergic and glutamatergic MLR cells collectively control decelerating and accelerating locomotor behaviors. Work in cats shows that the substantia nigra pars reticulata (SNr) projects to the PPN and suppresses muscle tone while another projection from the lateral SNr to the CnF promotes locomotor activity (Takakusaki et al., 2003). It is thought that the cholinergic MLR population modulates locomotion, but activation of these cells is not sufficient to elicit a locomotor bout. Instead, stimulation of cholinergic MLR cells leads to acceleration of locomotion (Roseberry et al., 2016). However, there are interspecies differences in the density of cholinergic cells. For example, in Parkinsonian patients where the number of cholinergic cells in the PPN are reduced (Hirsch et al., 1987; Jellinger, 1988; Zweig et al., 1989), gait disturbances are often observed (reviews Pahapill and Lozano, 2000; Alam et al., 2011). In addition, basal ganglia afferent connectivity onto the PPN, and from the PPN onto the basal ganglia differ between species. This may help explain the uncertainty in the efficacy of deep brain stimulation stimulation in Parkinson's patients (Alam et al., 2011). Some studies have reported beneficial effects but other studies are less supportive. There is a debate about whether stimulation is targeting the PPN, with the most effective site being slightly posterior to the PPN, in the CnF and the subCnF (Ferraye et al., 2010). Notably the location of the nuclei encompassing the MLR is still a matter of debate, and there appears to be interspecies differences (for details refer to Alam et al., 2011, 2013; Liang et al., 2011, 2012; Thankachan et al., 2012; Ryczko and Dubuc, 2013; Xiang et al., 2013; Sherman et al., 2015). The CnF is an important part of MLR in higher mammals such as cats and monkeys, whereas the precuneiform nucleus (PrCnF) is the mouse analog of CnF. In mice the PrCnF projects directly to the spinal cord (Liang et al., 2011, 2012). This is not the case in the cat where the CnF was found to project to the first cervical segment. Likewise, in monkeys, the CnF projects ipsilaterally within segments of the spinal cord. An additional complexity in interpreting the literature is that the size of the PrCnF in cats (Satoda et al., 2002) and possibly monkeys (Castiglioi et al., 1978) is likely underestimated as the boundaries between the PrCnF and CnF in cats are not as distinct (Liang et al., 2011).

To summarize, the MLR is well-studied, but comparing and contrasting studies, especially between species can be difficult. Generally speaking, in rodents, the data suggest that the PPTg (PPN) is better associated with reward-based motor behaviors, place preference, and sensorimotor gating, than locomotion (Koch et al., 1993; Inglis et al., 1994; Olmstead and Franklin, 1994; Alam et al., 2011). On the other hand, the CnF and PrCnF seem to be better associated with locomotion (Garcia-Rill and Skinner, 1987a; Milner and Mogenson, 1988; Noga B. et al., 2017).

## The diencephalon: a hub for goal-directed locomotion

The diencephalon is home to several regions that can elicit locomotion. Although, diencephalic sites have been described in several species, they have been named differently based on the differences in anatomy and effective sites for stimulation. A region of the diencephalon that was pro-locomotory was first described in the cat in the 1930's (Ectors et al., 1938; Masserman, 1938), and electrical stimulation of the subthalamic region was conducted by Waller (1940). Additional work in the 1980s from Orlovsky, Sinnamon, and Mori amongst others provided new insight into areas within the posterior hypothalamus, lateral hypothalamus, and zona incerta that could elicit locomotor activity. While differences were observed, a general finding was in freely moving animals, it was found that an initial scan of the area was performed before exploratory activity was initiated (Mori et al., 1989). These behaviors are often indistinguishable from spontaneous locomotor behavior (Grossen and Kelley, 1972; Leppänen et al., 2006; Lamprea et al., 2008).

Initial reports suggested the site for eliciting locomotion in the cat was the subthalamic nucleus (STN) (abbreviated as STh in rodents); and thus, it was named the subthalamic locomotor region (SLR) (Grossman, 1958; Kaelber and Smith, 1979). Work conducted later in the rat found that the zona incerta (or “zone of uncertainty” dorsal to STh) and medial lateral hypothalamus (LH) had lower thresholds for electrical stimulation (Figure 1; Sinnamon and Stopford, 1987; Milner and Mogenson, 1988; Sinnamon, 1993). In the lamprey, this region is known as the diencephalic locomotor region (DLR) (Manira et al., 1997; Ménard and Grillner, 2008), and is located ventral to the thalamus—a region analogous to the lateral hypothalamus in mammals (Ménard and Grillner, 2008). Unfortunately, like the MLR, the terminology used over the years has been confusing. Here we will use the historic SLR term but, where possible, we'll specify the anatomical region.

The SLR is necessary for goal-directed locomotion as bilateral ablation of the SLR abolishes spontaneous locomotion for several weeks following surgery (Shik and Orlovsky, 1976—original article in Russian: Sirota and Shik, 1973). Although, the SLR is connected to the MLR, the MLR is not necessary for SLR-evoked locomotor behavior (Shik et al., 1969). It appears from these findings that the SLR serves as a parallel command system for locomotor control. What remains unclear is if the MRF is a necessary integration center to spinal cord to produce locomotion. In the lamprey, the DLR, an analog of the SLR in fish, projects to reticulospinal cells (Manira et al., 1997). However, in mammals, direct descending projections from the SLR and DLR to the spinal cord have been reported (Skagerberg and Lindvall, 1985; Sakurai, 2005; Stoyanova et al., 2010; Koblinger et al., 2014). Therefore, it is possible that an MRF-relay may not be necessary for all locomotor behaviors. A more likely scenario is that parallel pathways converge on spinal circuits to coordinate most behaviors.

Local infusion of glutamate agonists and GABA antagonists into the zona incerta (ZI) or the LH are sufficient to drive locomotor behaviors, suggesting that both inhibitory and excitatory afferents regulate SLR output (Di Scala et al., 1984; Milner and Mogenson, 1988; Sinnamon, 1993). The cell types of the ZI and LH have been well-characterized, and like other locomotor command centers are composed of heterogeneous cell types. These include fast-transmitting GABA, glutamatergic cells, and various peptidergic and neuromodulatory subtypes (review Mitrofanis, 2005; Stuber and Wise, 2016). Two modulatory systems that robustly drive locomotion are orexin (Valenstein et al., 1970; Valenstein, 1971; Ida et al., 1999; Thakkar et al., 2001; Sakurai, 2005; Siegel and Boehmer, 2006) and dopamine (Wagner et al., 1995; Kolmac and Mitrofanis, 1999).

The orexin cells in the brain originate mainly in the LH and activity of orexinergic cells is tightly coupled to the regulation of arousal, sleep, appetite, attention, and sensory modulation (Valenstein et al., 1970; Valenstein, 1971; Ida et al., 1999; Thakkar et al., 2001; Sakurai, 2005; Siegel and Boehmer, 2006). These cells have extensive projections throughout the brain including, but not limited to, the SNr, MLR, MRF, and spinal cord (Peyron et al., 1998; Sakurai, 2005; Stoyanova et al., 2010). Mice exhibit enhanced locomotor activity following intracerebroventricular administration of orexin (Hagan et al., 1999; Ida et al., 1999), and blocking orexin-1 receptors attenuates movement (Duxon et al., 2001). A recent study showed presence of orexin receptors on the reticulospinal MLR cells (Sherman et al., 2015). Moreover, focal injection of orexin into the MLR in decerebrate cats either reduced the intensity to evoke locomotion or elicited locomotion without stimulation, whereas an injection of orexin in either PPTg or SNr increased the intensity required to induce muscle atonia (Takakusaki et al., 2005). Recent work has reported that orexin cells may drive locomotor activity by increasing the activity of glutamic acid decarboxylase (GAD65)—expressing inhibitory neurons located in the LH. This population of inhibitory cells was found to be able to drive locomotor behavior when activated, and suppress locomotion when inhibited. These GAD65-expressing cells send projections to ZI, raphe magnus as well as superior colliculus and periaqueductal gray (Kosse et al., 2017; see section Interactions between Appetitive, Defensive, and Exploratory Behavior); however, the underlying circuitry responsible for orexinergic locomotor control requires further characterization.

The A13 and A11 dopaminergic nuclei are in the ZI and the rostral portion of the posterior hypothalamus, respectively. The contribution of the A11 to locomotor control is well characterized in zebrafish and is known as the diencephalospinal dopamine system (DDS; Tay et al., 2011; Lambert et al., 2012). The most recent work on the DDS demonstrates that these cells are rhythmically active during swimming and are both sufficient and necessary for swimming episodes (Jay et al., 2015). Currently, little is known about the locomotor functions of the A13 and A11 in mammals. However, the A11 projects to the spinal cord (Commissiong and Sedgwick, 1975; Skagerberg and Lindvall, 1985; Holstege et al., 1996; Koblinger et al., 2014; review Sharples et al., 2014) and has known roles in pain modulation (Charbit et al., 2009) and motor control (Ondo et al., 2000; Clemens et al., 2006; Qu et al., 2007). It has also been shown that dopamine modulates mammalian spinal CPG networks (Barrière et al., 2004; Humphreys and Whelan, 2012; Sharples et al., 2015; Picton et al., 2017; Sharples, 2017; Sharples and Whelan, 2017). These data support a possibility for a descending dopamine system in mammalian locomotor control. Nonetheless, the role of A11 and A13 cell populations in locomotor behaviors remains to be tested.

## Factors mediating decision making and motor selection to approach or to avoid

**Figure 2:**
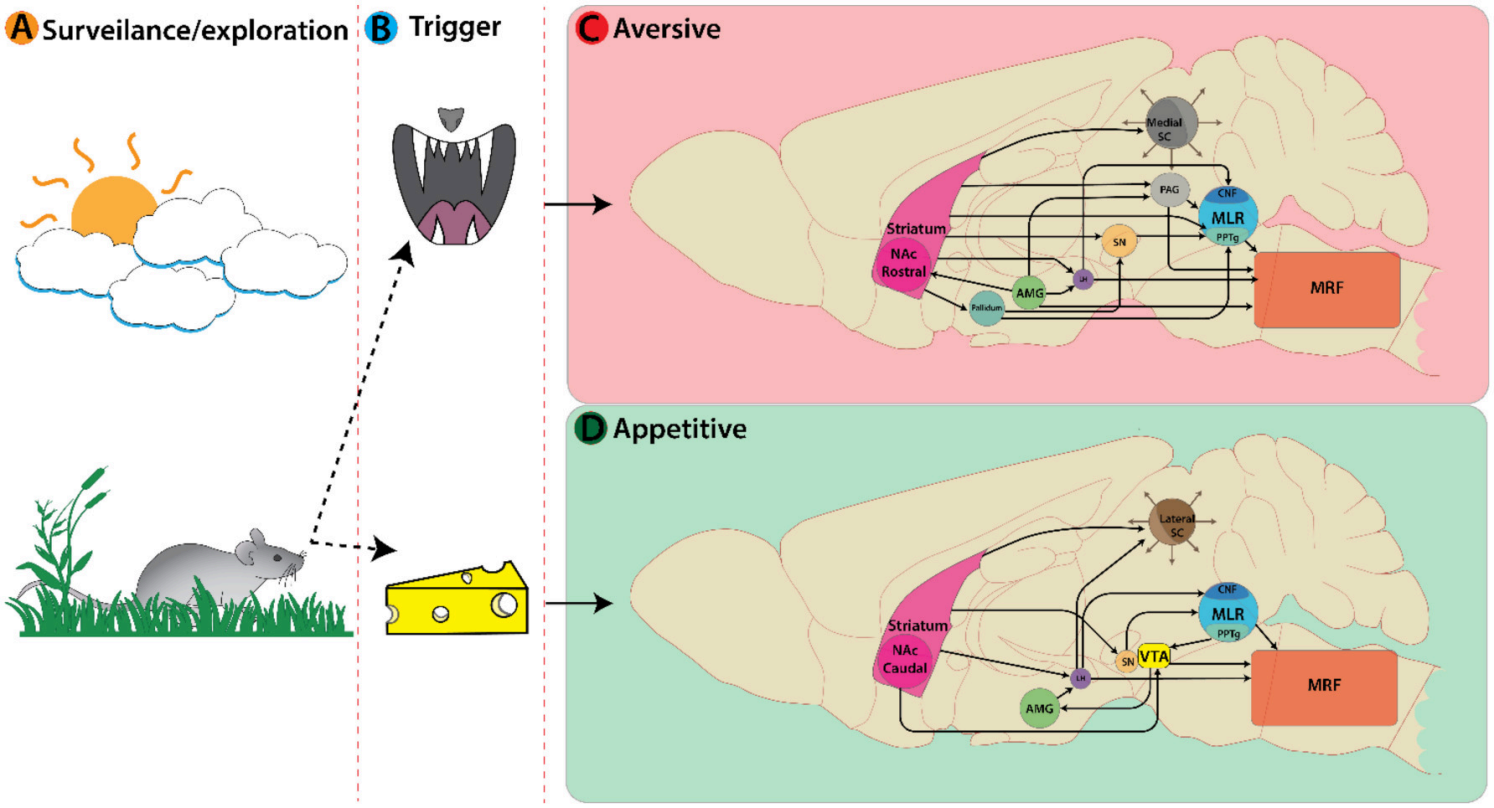
Active nuclei in the mouse brain following a decision or trigger from a surveillance state. **(A)** Representation of an animal's decision process during a surveillance state. In this example a mouse encounters an aversive trigger (**B** top, predatory threat) activating nuclei associated with avoidance behaviors **(C)**. Main nuclei active here are: Striatum, Amygdala, Superior colliculi, etc. On the bottom **(B)** the mouse encounters an appetitive trigger of food/cheese activating nuclei associated with approach behaviors **(D)**.

So far, we have discussed locomotor regions by illustrating the nuclei and cell types involved in each pathway. These regions must interact with other brain areas to produce behaviorally relevant locomotion. Broadly speaking, there are two basic forms of locomotor responses observed in vertebrate species—approach or avoid—and a balance between these is necessary for survival (Glickman and Schiff, 1967). Here we will describe how internal (affective state, cognitive, reward, motivation, homeostatic, etc.) and external (sensory) cues are integrated to decide on approach or aversion (Figure 2A). We will describe the functional role of different types of locomotion based on their behavioral correlates, with emphasis on: (1) the role of the superior colliculus (SC) in deciding the appropriate locomotor response based on external sensory cues; (2) key limbic structures mediating locomotor responses based on internal cognitive and affective information; and (3) subsequent motor selection via the basal ganglia circuitry.

## External sensory cues can facilitate appropriate motor selection

External sensory cues are broadly classified as olfactory, visual, auditory, and tactile stimuli. When animals encounter such cues they must react appropriately to meet survival needs (Figure 2B).

The SC is an integral player in triggering appropriate locomotor responses based on novel visual stimuli in the environment. The superficial layers of the SC make use of retinotopic information relayed via the lateral geniculate nucleus of the thalamus (Nagata and Hayashi, 1984; Born and Schmidt, 2008) and primary visual cortex to orient the eyes, head, and body movement toward objects of interest (reviews Sparks, 1986; Grillner et al., 2008; Gandhi and Katnani, 2011). This permits the SC to trigger approach or avoidance responses based on visuospatial input. The location of visual information within the visual field is key for identifying visual cues as food or a threat. Presenting an approaching visual stimulus in the upper visual field can evoke defensive responses such as escape and freezing in mice (Yilmaz and Meister, 2013). In rodents, predatory visual input is in the upper visual field and mapped in the medial SC (Figure 2C), whereas appetitive stimuli are detected in the lower visual field and mapped in the lateral SC (Comoli et al., 2012; Figure 2D). This functional organization is reflected by increased c-Fos protein expression (sign of recent neural activity) in the lateral SC following a hunting session for roaches on the floor (Favaro et al., 2011). In addition, unilateral electrical stimulation of lateral SC elicits contralateral orienting and approach-like responses, while stimulation of medial SC induces ipsilateral cringe-like defensive movement that develops into locomotion, running, and jumping with increasing stimulation intensity (Sahibzada et al., 1986). Such orienting responses involve contralateral MRF pathways, whereas movement away from the stimulus is mediated exclusively by an ipsilateral MRF pathways originating from the ventral and lateral SC (Sparks, 1986).

The SC projects ipsilaterally to the CnF and contralaterally to PPTg (Dean et al., 1989) with a major projection onto GABAergic MLR cells (Roseberry et al., 2016). The progression of locomotion elicited by stimulation of the medial SC is like that observed following stimulation at the MLR (Shik et al., 1969; Roseberry et al., 2016). One possibility is that this projection is inhibitory in nature, allowing for locomotion to occur via disinhibition of the MLR. Alternatively, if this circuit is glutamatergic, activation of GABAergic MLR neurons could suppress glutamatergic MLR output, producing motor arrest (see section Reflexive Startle Response Driven by Sudden External Sensory Stimuli).

## Decisions to approach or avoid are guided by internalized contextual information

Generally, animals approach rewarding stimuli and avoid aversive stimuli. Aside from the positive and negative values associated with external stimuli, the coordination of approach- or aversive-like behaviors depend on the animal's internal affective and motivational states (Loewenstein et al., 2015). In early studies, Denny-Brown (1962) showed that bilateral lesions of the striatum caused animals to follow anything that moved. Since then the limbic system is understood to contribute to context-specific locomotion that drive decisions to approach or avoid.

Within the limbic system, the nucleus accumbens (NAc) of ventral striatum is an important limbic-motor interface underlying reward and motivation states (Mogenson et al., 1980; Roitman et al., 2005; Carlezon and Thomas, 2009; Levita et al., 2009; Humphries and Prescott, 2010; Richard and Berridge, 2011; McCutcheon et al., 2012; Salgado and Kaplitt, 2015). For example, amphetamine injected in the NAc results in hyper-locomotion demonstrating the key role of dopamine in both locomotion and reward. The NAc is divided into two subregions: the shell and the core. Traditionally, the shell of the NAc orchestrates the response to unconditioned, innate reward and indeed lesions of the shell produce hypolocomotion (Ito et al., 2004; Aragona et al., 2008; Ito and Hayen, 2011). On the other hand, the core mediates approach behaviors associated with Pavlovian reward-associated cues (Parkinson et al., 2000; Ito et al., 2004; Stefanik et al., 2013, 2016; Hamel et al., 2017). The NAc has diverse outputs that enable recruitment of locomotor circuits. The NAc recruits locomotor circuitry via projections to: globus pallidus (GP), substantia nigra pars compacta (SNc), SNr, LH, ventral tegmental area (VTA), periaqueductal gray (PAG), PPTg, and ventral pallidum (VP) (Mogenson et al., 1983; review Nicola, 2007). It may be noted here that in some of these experiments PPTg also included overlapping brain regions including the CnF. Together these outputs explain the strong locomotory effect of stimulation of the NAc [sections Locomotor Response in Anticipation of Reward and Defensive Locomotor Responses (Escape and Freeze) Associated with Aversive Cues].

The amygdala is implicated in decisions regarding approach and avoidance locomotor behaviors (Petrovich, 2011). Learned food cues are relayed to the LH from the basolateral amygdala complex (BLA) to facilitate feeding, and aversive cues can suppress feeding by direct and indirect projections from the central nucleus of the amygdala (CeA) to LH (Petrovich et al., 1996; Petrovich, 2011). Differential modulation by the CeA and BLA onto striatal projection neurons is known and thus can bias approach and avoid behavior selection (Wall et al., 2013). Discrete nuclei within the amygdala have direct and indirect projections to the PAG, which are involved in defensive locomotion (Gross and Canteras, 2012). Although, the amygdala is involved in both approach and avoidance, its role in driving the corresponding locomotor response to associated triggers requires interactions with various brain regions. The role of the amygdala in both appetitive and aversive locomotor behavior will be revisited separately in the following sections (sections from Motivation to Approach: Execution of Forward Locomotion and Reflexive Startle Response Driven by Sudden External Sensory Stimuli).

## Motor selection derived from the decision to approach or avoid

Both external and internal cues interact with basal ganglia (BG) circuits for precise execution and selection of appropriate locomotor behavior. One widely accepted hypothesis is that the direct dopamine pathway facilitates reward-oriented motor behavior (reviews Everitt and Robbins, 2013; Kim and Hikosaka, 2015; Grillner and Robertson, 2016; Averbeck and Costa, 2017) while the indirect pathway suppresses unrewarded movements. Hence, these two pathways may regulate most associative learning and reward-oriented motor actions (Frank, 2006; Kravitz et al., 2010; Hong and Hikosaka, 2011). The two pathways originate from GABAergic striatal projection neurons that are known as the direct and indirect pathway medium spiny neurons, dMSNs, and iMSNs, respectively (review Utter and Basso, 2008). The dMSNs which express dopamine D1 receptors are excited by dopamine and project directly to the output nuclei of the basal ganglia, the SNr and GPi. Tonically active GABAergic projection neurons comprise these output nuclei and are responsible for tonic inhibition of the thalamus, SC, and PPTg. Thus, inhibition of GPi/SNr neurons by GABAergic dMSNs leads to disinhibition of brainstem motor centers and allow movement initiation. In contrast, iMSNs express dopamine D2 receptors and are inhibited by dopamine. These send GABAergic projections to the globus pallidus external (GPe) which in turn projects inhibitory output to the GPi. Sequentially, the GPe sends inhibitory output to excitatory STh that targets the SNr. Therefore, the net effect of the indirect pathway is an enhancement of inhibitory input from GPi and SNr to the descending motor centers. The GPi/SNr projects to the PPTg providing tonic inhibition affecting locomotor behavior. The anatomical tracing studies provide evidence for SNc projections to the PPN in rat (Beckstead et al., 1979; Semba and Fibiger, 1992; Steininger et al., 1992; Ichinohe et al., 2000) and in cat (Edley and Graybiel, 1983). The presence of such descending input was also supported by recordings of short latency antidromic activation of SNc neurons following PPN stimulation in rat (Scarnati et al., 1984, 1987). This is important as it suggests that dopamine has exclusive projections to brainstem nuclei distinct from the BG circuitry (Ryczko and Dubuc, 2013; Ryczko et al., 2016), which adds an extra degree of monoaminergic control over movement initiation.

## Primary appetitive locomotor system: approach-like responses to rewarding & appetitive cues

**Figure 3:**
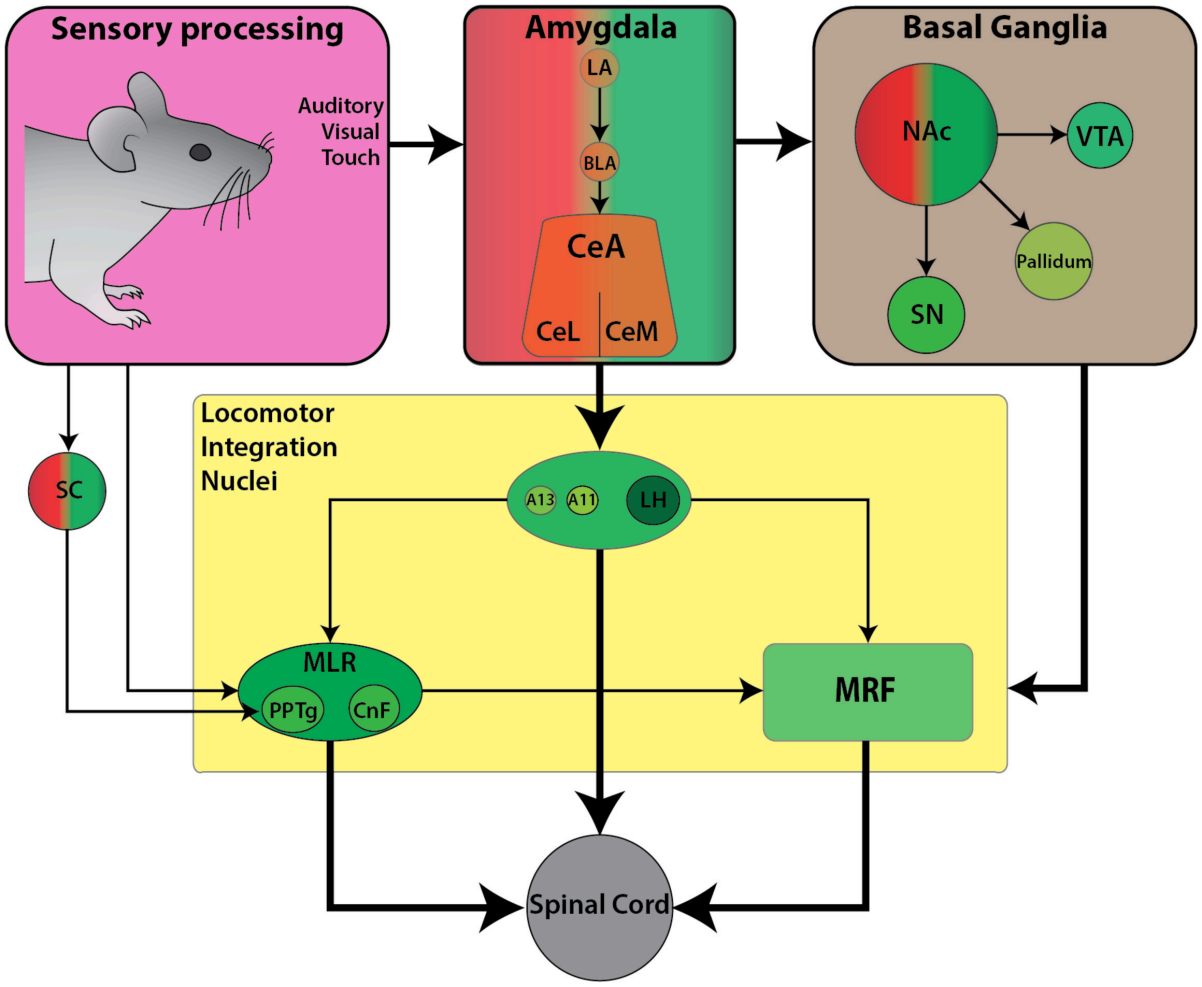
Schematic diagram of the currently known descending connectivity of appetitive (approach) locomotor control. (Complementary to Figure 2D. Some connections omitted for lack of data or complexity of connectivity). Weighted arrows represent complex connectivity between regions described in further detail in text. Ascending connectivity has been omitted for clarity.

Internalized contextual information such as reward and motivation combine to form approach-like behaviors across species. For example, place preference and self-stimulation paradigms often require association with rewarding cues, such as food to facilitate subsequent approach responses toward stimuli. Here we will examine work focused on how locomotion toward a rewarding stimulus is achieved. We will describe the functional connectivity between the limbic circuitry and descending locomotor centers, relevant for mediating forward locomotion in the context of appetitive behaviors (Figure 3).

## From motivation to approach: execution of forward locomotion

The motivation to approach a stimulus in the environment stems from associations between the stimulus and its value as a physiological need or reward. Limbic structures such as the amygdala are key in mediating an approach locomotor response toward a rewarding stimulus such as food. As mentioned in section Decisions to Approach or Avoid are Guided by Internalized Contextual Information, the amygdala (BLA) directly projects to the LH which is a key hub for appetitive behaviors. The LH is an excellent candidate for investigating how reward-related information integrates with descending locomotor centers to facilitate forward locomotion toward reward-associated stimuli.

The LH is a large structure and has multiple functions. In the locomotor field, the initial descriptions of the LH were studied in the context of appetitive locomotor control (Sinnamon, 1993). However, most recent work has been directed toward the role of the LH in modulating homeostatic demands. Electrical stimulation of the LH elicits diverse responses beyond locomotion, such as feeding, drinking, gnawing and predatory attack that often vary from animal to animal but are linked to the current external stimuli (Coons et al., 1965; Roberts and Carey, 1965; Mogenson and Stevenson, 1967; review Stuber and Wise, 2016). Several LH cell populations have been shown to play a key role in feeding and appetite regulation including orexin, GABAergic, and glutamatergic cells. Projections onto the LH are predictably diverse and include olfactory and pyriform cortex, NAc, dorsal striatum, GP, ZI, perifornical region, most hypothalamic areas including magnocellular and medial preoptic, supraoptic, paraventricular and periventricular nuclei, posterior hypothalamus, arcuate and mammillary nuclei, bed nucleus of stria terminalis, ventral thalamic nuclei, VTA, SN, MRF, PAG, locus coeruleus, and parabrachial region (Barone et al., 1981).

The LH orexinergic transmission plays a key role in mediating locomotor responses via SN and brainstem locomotor regions (section The Diencephalon: A Hub for Goal-Directed Locomotion) and are active during feeding behavior (de Lecea et al., 1998; Sakurai et al., 1998), reward and arousal (Peyron et al., 1998; Baldo et al., 2003; Harris et al., 2005; Swanson et al., 2005; Aston-Jones et al., 2010). Indeed, the orexinergic neuronal population shows the highest level of spiking activity when animals are moving toward a food source. Orexinergic neurons are not the only LH neurons involved in locomotion; recently GABAergic LH cells have been reported to contribute to modulate locomotor activity. Chemogenetic silencing of LH GABAergic cells depresses voluntary locomotion, while stimulation leads to hyper-locomotion (Kosse et al., 2017). Anterograde tracing of these GABAergic cells uncovered substantial projections onto the ZI. The downstream projections are unknown. These ZI cells could also be modulated by orexin since photostimulation of orexinergic cells rapidly recruits GABAergic LH cells, and spiking of these GABAergic LH cells precedes spontaneous running bouts (Kosse et al., 2017).

## Locomotor response in anticipation of reward

While motivation contributes to an animal's approach behavior toward rewarding stimuli (Mogenson et al., 1980), anticipation of reward can also drive forward locomotion. Such anticipatory reward signals are integrated to locomotor centers via corticolimbic structures including the cortex, striatum and pallidum, which have descending projections to the locomotor regions (Swanson, 2000).

As well as participating in motor selection (section Motor Selection Derived from the Decision to Approach or Avoid), dopamine circuits including mesolimbic, mesocortical, and nigrostriatal pathways govern reward-related behaviors. Dopamine release within the NAc is an important determinant of reward processing. Furthermore, NAc is known to have reciprocal projections to dopaminergic neurons in VTA and the SN which projects to the dorsal striatum (Haber et al., 2000; Ikemoto, 2007). The dorsal striatum and its dopaminergic inputs serve key roles in the regulation of locomotor control (Faure et al., 2005; Belin and Everitt, 2008; Palmiter, 2008). Striatal pathway projections are differentially modulated by dopamine, and are either excitatory via the direct pathway (D1 receptor) or inhibitory via the indirect pathway (D2 receptor) (Surmeier et al., 2007). The locomotor modulation observed within BG circuitry could occur via glutamatergic and cholinergic MLR neurons that mediate initiation and acceleration of locomotion while GABAergic populations could facilitate deceleration, respectively (Roseberry et al., 2016). Dopaminergic fibers have been reported around cholinergic cells in MLR of lamprey (Ryczko et al., 2013), salamander (Ryczko et al., 2016), rat (Ryczko et al., 2016), monkeys (Rolland et al., 2009), and human (Ryczko et al., 2016) indicating that the innervation of the MLR is conserved in vertebrates (Ryczko and Dubuc, 2017). In lamprey and salamander, the origin of this dopaminergic innervation to cholinergic cells in MLR was found to be a diencephalic dopaminergic region termed as posterior tuberculum which sends ascending projections to the striatum and is considered homologous to mammalian SNc and/or VTA (Yamamoto and Vernier, 2011; Wullimann, 2014; Ryczko et al., 2016; Ryczko and Dubuc, 2017). While only a few dopamine neurons sent collaterals to the striatum and the MLR in lampreys and salamanders, numerous SNc dopamine neurons have both ascending and descending collaterals in rats (Ryczko et al., 2016). The number of ascending dopaminergic collaterals may be related to evolutionary expansion of the basal ganglia (Grillner and Robertson, 2016; Ryczko and Dubuc, 2017). These findings suggest that the role of dopaminergic activity in reward-related behavior is bidirectional and could occur in anticipation of obtaining a reward.

Following a similar theme, neurons within the PPTg are important for modulating speed and gait during locomotion (section The Mesencephalic Locomotor Region: An Integrative Hub for Locomotor Speed and Gait), but also respond in anticipation of reward signals. PPTg neurons respond phasically to auditory and visual sensory stimuli that predict reward with a shorter latency (5–10 ms) than dopaminergic VTA/SNc cells (Pan and Hyland, 2005). Furthermore, Norton et al. (2011) examined PPTg neural activity as rats solved a spatial working memory task that involved retrieving rewards of different magnitudes from known locations. Interestingly, they reported separate populations of PPTg neurons independently code for reward or movement. Thus, the reward anticipatory response within PPTg is part of a feedforward mechanism to trigger a fast locomotor response triggered by a reward-associated cue. In support of this idea, photoactivation of cholinergic PPTg terminals at SNc has been shown to increase locomotion (Xiao et al., 2016). Electrical stimulation of PPTg induces a burst firing of midbrain dopaminergic neurons (Lokwan et al., 1999; Floresco et al., 2003) with concomitant release of dopamine in striatum (Chapman et al., 1996; Miller and Blaha, 2004). This suggests that the PPTg may facilitate feedforward and feedback loops with SNc via reciprocal projections. Calcium transients within dopaminergic terminals in dorsal striatum precede (100–150 ms) bouts of locomotion independent of reward expectation (Howe and Dombeck, 2016). Photoactivation of these dopaminergic axons leads to initiation of locomotion bouts. Thus, rapid sub-second phasic signaling contributes to locomotion bout initiation associated with ongoing accelerations. Since glutamatergic MLR cells can facilitate acceleration (Roseberry et al., 2016), the role of these populations in potentiating locomotion in anticipation of reward is of interest.

## Primary defensive locomotor system: avoidance responses to aversive associated cues

**Figure 4:**
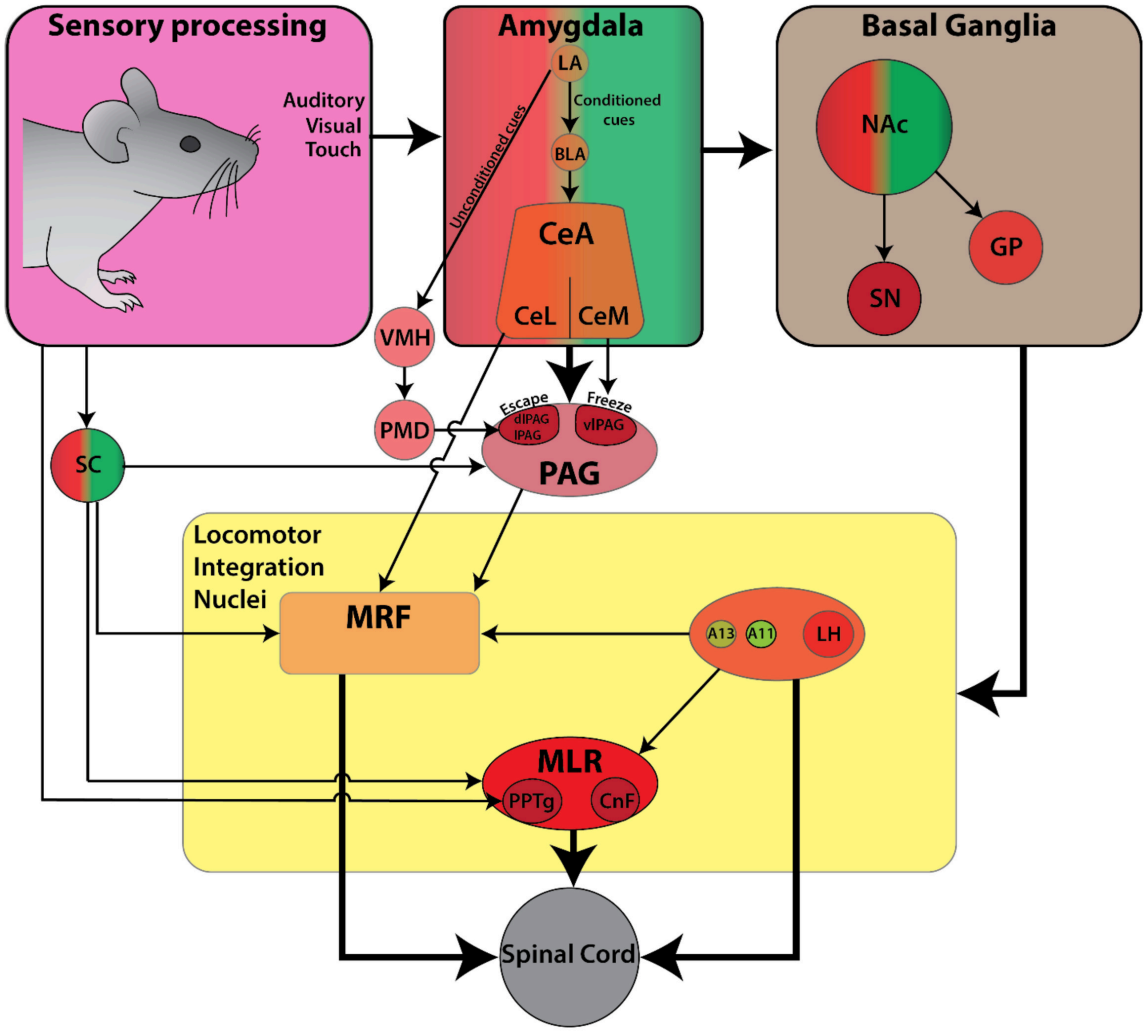
Schematic diagram of the currently known descending connectivity of defensive (avoidance) locomotor control. (Complementary to Figure 2C. Some connections omitted for lack of data or complexity of connectivity). Ascending connectivity has been omitted for clarity.

Locomotion is a critical element in the primary defensive system since it serves to increase the distance away from threatening or painful stimuli (Sinnamon, 1993). In general, aversive behaviors are characterized by three types of responses: (1) a reflexive startle response, (2) escape behavior to flee from an aversive stimulus, and (3) a freezing response. Of these three defensive responses, the circuitry for startle response has been best described. On the other hand, escape and freezing responses have often been used as behavioral outcomes in pain and fear related studies, but the associated locomotor components have received less attention (reviews Klemm, 2001; Roseberry and Kreitzer, 2017; Figure 4).

## Reflexive startle response driven by sudden external sensory stimuli

The mammalian startle response is characterized by fast twitch of facial and body muscles as well as an arrest of ongoing movement in response to a sudden and intense sensory stimulus (Koch, 1999). It protects the animal from predation by preparing for a flight or fight response, or by freezing so the animal can't be easily seen (Landis and Hunt, 1939). A startle response can be elicited by different sensory modalities which act via separate pathways. These include: (1) trigeminal for sudden tactile stimuli, (2) auditory for sudden acoustic stimuli, and (3) vestibular for sudden head movements. Though these startle pathways originate from different second-order afferents (nucleus V for tactile, cochlear root nucleus for auditory, and vestibular nucleus for balance), they converge onto giant neurons in the PnC projecting directly to motoneurons and interneurons of the spinal cord (reviews Koch, 1999; Yeomans et al., 2002). Less well understood is the light-induced startle response characterized by temporary locomotor arrest following brief flashes of light (Liang et al., 2015). This phenomenon can be partly driven by input to the SC from layer 5 of the primary visual cortex (V1 L5). Photoactivation of V1 L5 neurons projecting to SC directly triggers locomotor arrest in running mice (Liang et al., 2015). Inactivating the SC with muscimol reduces the arrest behavior by 76%, whereas silencing V1 by optical activation of parvalbumin-positive inhibitory neurons reduces arrest by 33% (Liang et al., 2015). Overall, these startle pathways are fine-tuned to detect specific triggers from the environment during the default exploration state of the animal, allowing an animal to quickly transition into a defensive state (Yeomans and Frankland, 1995).

## Defensive locomotor responses (escape and freeze) associated with aversive cues

In comparison to the startle response, which is reflexive in nature, the choice between escaping a stimulus and freezing is highly dependent on contextual cues. Behavioral outcomes that measure fear and pain such as conditioned place preference/aversion and dynamic weight bearing analysis rely on quantification of locomotor behavior. The descending control of locomotor circuits in response to aversive stimuli has not been fully explored. Nonetheless, aversive responses rely heavily on locomotor circuit interactions between the amygdala and PAG (LeDoux, 2012; Koutsikou et al., 2015, 2017).

Aversive stimuli can be thought of as either unconditioned and innate, or learned and associated with pain and fear. The association between the unconditioned stimulus and the conditioned stimulus required may involve memory processing related to fear. This is mediated in part by both the CeA and BLA of the amygdala (LeDoux, 2000; Medina et al., 2002; Wilensky et al., 2006; Ciocchi et al., 2010; Duvarci et al., 2011; Li et al., 2013; Han et al., 2015; Sato et al., 2015). The CeA is an important neural substrate for the expression of the freeze response (Davis and Whalen, 2001; Phelps and LeDoux, 2005). It projects to the hypothalamus, dorsal and ventral striatum, PAG, and MRF, and could also modulate cognition in a broader sense, via its outputs to ascending monoaminergic and cholinergic systems such as: noradrenergic LC, dopaminergic SNc and VTA, serotonergic raphe, and the cholinergic nucleus basalis (Davis and Whalen, 2001; Sara, 2009). Recent cell-type-specific viral tracing studies have also revealed a strong projection from the CeA to glutamatergic MLR neurons, which can initiate locomotion from rest when activated (Roseberry et al., 2016). Similarly, chemical or electrical stimulation of CeA can elicit freezing or fleeing behavior (Brandão et al., 1999, 2015; Vianna et al., 2001; Muthuraju et al., 2016). Thus, the CeA is important for orchestrating defensive freeze and escape responses (Sah et al., 2003; Oka et al., 2008).

The PAG also plays a central role in regulating defensive locomotor behaviors. Bandler was the first to show the PAG's direct role in defensive reactions in cats by pharmacological activation with microinjections of glutamate (Bandler, 1982) and excitatory amino acids (Bandler and Carrive, 1988). Subsequent studies identified five subregions that can be differentiated based on anatomy, physiology, and behavioral outcomes when activated (Carrive, 1993). The dorsolateral/lateral (dl/l) and ventrolateral (vl) columns of the PAG appear to be the most relevant in the context of defensive locomotor behaviors. Activation of the lPAG can elicit a variety of responses such as: strong hindlimb movements for flight reaction, reactive immobility accompanied by heightened responsiveness to surrounding stimuli, backward locomotion, and forward escape locomotion with occasional jumps (Bandler and Depaulis, 1988; Depaulis et al., 1989, 1992; Carrive, 1993). Meanwhile, activation of the vlPAG can induce hyporeactive immobility characterized by reduced spontaneous activity and/or responsiveness to surrounding stimuli (Bandler and Depaulis, 1988; Depaulis et al., 1992; Carrive, 1993). In line with this, electrolytic lesions of vlPAG in rats decrease freezing induced by unconditioned and conditioned stimuli, whereas lesions of dlPAG enhanced freezing (Fanselow et al., 1995; De Oca et al., 1998). Photoactivation of glutamatergic cells of the dorsal PAG (dPAG) can evoke both freeze and escape behavior in a gradual manner via firing rate and temporal coding mechanism (Chen et al., 2015). Increasing either frequency or intensity of photoactivation progressed defensive phenotype from freeze to escape and then to jump. Given the lack of a clear boundary between observed phenotypes, it is possible that these cells may perform this function through differing neurotransmitter release and/or its afferent and efferent connections.

Sensory cues that trigger aversive behaviors are either unconditioned or conditioned, and are processed through different pathways. Unconditioned odor cues are conveyed via the medial amygdala, whereas auditory and visual cues are conveyed via the accessory basal amygdala. These signals are further processed through the circuitry of the ventromedial hypothalamus (VMH)-premammillary nucleus of the hypothalamus (PMH) and the dPAG. On the other hand, conditioned cues are processed via the LA and intra-amygdala connections onto CeA, and then project onto vlPAG from medial CeA (CeM) (Motta et al., 2009). The CeA also projects directly onto PnC (Davis and Whalen, 2001) where Chx10 stop neurons could be found (Bouvier et al., 2015) and glutamatergic MLR neurons which are associated with acceleration of ongoing movement (Roseberry et al., 2016). Therefore, defensive responses may be mediated through these projections as well. Furthermore, glutamatergic cells within PAG innervate the GiA, GiV, and LPGi (Tovote et al., 2016). Most work has focused primarily on fear induced freezing; however, the neural circuitry of PAG-mediated defensive locomotor control remains undefined. Considering this, Tovote et al. (2016) selectively manipulated inhibitory projections from the CeA to local inhibitory interneurons within the vlPAG. Glutamatergic neurons of the vlPAG are under local inhibition, and upon disinhibition by the CeA, they induce freezing via GiA, GiV, and LPGi targets. In contrast to activation of glutamatergic vlPAG neurons, photostimulation of glutamatergic dl/lPAG neurons evoke bouts of escape locomotor behavior intermingled with short freezing periods (Tovote et al., 2016). It seems that the PAG could play an important role in translating locomotor behaviors related to freezing or escape and may interact with locomotor command centers. A few studies suggest that an interaction between PAG and locomotor command centers (e.g., CnF) may be relevant for locomotor behaviors in freezing and/or escape (Ferreira-Netto et al., 2007). Connectivity between PAG and Chx10 neurons within caudal PnC and rostral Gi which can evoke stop has not yet been explored. With these multiple possible points of integration between the pain, limbic, and motor systems that have been found through tracing techniques, we can begin to create testable models of functional circuitry within specific contexts that modulate locomotion.

## Interactions between appetitive, defensive, and exploratory behavior

Appetitive, defensive and exploratory locomotor behavior are all necessary for survival. This is reflected at the neural systems level, where processes for these behaviors are closely intertwined along with circuits involved in maintenance of food and resources, defense, fluid balance, thermoregulation, and reproduction (Saper, 2006). Eating must often co-exist with the threat of predation for many species. The risks need to be weighed with the rewards and clearly, they are weighed differently in satiated compared to hungry animals. In the above sections, we have provided examples of nuclei that contribute to this complex predation calculus such as SC, NAc, and amygdala.

Since the locomotor responses are driven by the animal's interaction with the environment, there are multiple overlapping cognitive or associative and emotional processing structures modulating locomotor responses. Nevertheless, differential signaling processes for various contexts can facilitate approach while suppressing avoidance responses and vice versa. For example, GABA cells in the LH are excited by orexin and activity of these cells drives locomotion (section The Diencephalon: A Hub for Goal-Directed Locomotion). These inhibitory cells project to the SC and the PAG; both of which drive aversive locomotor behaviors. These inhibitory circuits are strong candidates for the suppression of aversive locomotor behaviors in situations where motivation to seek food is high.

In summary, innervation onto NAc could disinhibit the MLR via VP and the SNr to initiate, modulate or terminate locomotion. In addition, BLA and CeA send efferents to dMSNs preferentially (Roseberry et al., 2016) and the LH area (Petrovich et al., 1996; Petrovich, 2011), which could facilitate approach locomotion for cues associated with food and reward, or mediate suppression of appetitive behavior during defensive response and unrewarded movement, respectively. For example, in a behavioral conflict between aversion and reward, cholinergic and glutamatergic PPTg projections onto the dopaminergic VTA neurons may mediate a bias toward reward-oriented locomotion. Furthermore, PPTg projections to dopaminergic SNc neurons may facilitate suppression of unrewarded movements via the indirect pathway, or reinforce reward oriented motor action via direct pathway (Frank, 2006; Kravitz et al., 2010; Hong and Hikosaka, 2011). In addition, A11 and A13 (DLR) could provide a parallel dopaminergic modulation to mediate a balance between approach and avoidance behaviors.

## Future directions

This review has examined how locomotion is integrated into contextual demands. Recent advances in functional connectomics will enable us to decipher the circuits that underlie different approach and avoid motor behaviors. Specifically, optogenetics and chemogenetics coupled with viral-vector based approaches provide the tool kits necessary to advance our understanding. These methods can overcome limitations of electrical stimulation, and lesion approaches. That said direct comparisons with electrical stimulation remain useful since translational approaches using deep brain stimulation rely on this technology. In some cases, electrical stimulation may be advantageous since it's lack of selectivity may be required to adequately activate a network. Electrical stimulation will recruit more neurons due to failures in transfection. While optogenetics and chemogenetics are an excellent choice for understanding the connectome we need to carefully evaluate if these are the tool of choice for clinical use.

It is sobering to consider that establishing the connectome from an anatomical point of view is only half the story; the dynamic recruitment of multiple parallel pathways also needs to be considered. No matter which nuclei one focuses on there are multiple projections to other members within the overall circuit. We need to consider sampling key areas of the circuit simultaneously when designing studies so that we can begin to understand the dynamic recruitment of several centers during approach and avoidance tasks. These centers will include the PPTg, CnF, and MRF since they form key integrative centers in the brain. Depending on the type of study recordings need to be captured from the striatum, hypothalamic nuclei, and the LC.

## Challenges faced in driving contextual behavioral response

Meanwhile, as more sophisticated technologies and experimental tools became readily available, thus enabling the study of underlying mechanisms and circuitries associated with motor functions with much higher precision, there are several caveats. It is important to consider that the study of motor behaviors using experimental animal models generally require extensive training or conditioning, which rely on memory and learning processes. The impact of this training on the motor behavior being tested needs to be understood.

Learning and memory formation were initially thought to be driven by dynamic changes in synaptic strengths, which can be experimentally induced by high frequency stimulation (Bliss and Lomo, 1973). A few key regions of the brain were implicated, including the hippocampus, the neocortex, cerebellar, and brainstem nuclei (Medina et al., 2002). This is known as the synaptic plasticity and memory hypothesis, within this there are assumptions that synaptic inputs converge, or there are specific “relay centres” for information processing. This hypothesis has been reappraised because the mechanisms underlying the initial encoding and subsequent learning are likely to be different (Medina et al., 2002). This can be demonstrated by the observations that: (1) habituation occurs over relatively short periods of time for certain behaviors (e.g., forced swim, open field induced anxiety); and (2) neuronal plasticity often persists even when the conditioned behavior has fully extinguished (Hansel et al., 2001). The key point here is that whilst neuronal plasticity in specific regions contributes to the initiation of behavior, the maintenance may involve activity of other neuronal populations or pathways.

Similarly, the extinction of behaviors (e.g., fear extinction), indicate that learning is a state-dependent process. It was established that the amygdala, a brain region important for the regulation of emotion, receives input from the hippocampus and provides “context” (i.e., based on the animal's affective states) during conditioning (Phelps and LeDoux, 2005). In line with this review, modulation of locomotor behaviors is also context-dependent and are driven by the animal's need and/or adaptation to the ever-changing environments. At present, many studies investigating changes in cellular mechanisms or functional circuitry associated with motor behaviors are relatively short-term compared to behavior studies from other fields, such as sensory and cognitive neuroscience. Future studies must consider appropriate experiment paradigms to account for the changes in behaviors over time, and that there may be multiple changes at both cellular and systems levels in context-driven motor learning.

## Conclusion

To navigate through the environment, animals need to make on-the-fly adjustments to gait. Consider a baseball player running to catch a ball. The person will need to be motivated to run quickly to catch the ball, and this motivation may be higher at the World Series compared to a regular training day. Catching the ball requires visual input which is integrated through the dorsal stream to the motor cortex, which is then relayed back to diencephalic circuits including the BG and hypothalamic circuits. Modifications to gait need to be accomplished to execute accelerations, jumps, and slides. This is presumably integrated at the level of the MRF and MLR and integrated into ongoing activity within rhythm centers of the spinal cord. We are at a critical juncture in our understanding of how emotional and contextual cues affect locomotor performance. Many tools exist to tease apart circuit function in awake behaving animals and show how they affect downstream brainstem and spinal cord function. This of course dramatically increases the complexity of experiments directed at understanding motor control, but it will serve to highlight the interplay between regions of the brain involved in movement decisions that evolved to maximize survival of the organism.

## Author contributions

All authors (LK, SS, SAS, KM, CC, and PW) contributed to the conception, drafting, and critical revision of the review.

### Conflict of interest statement

The authors declare that the research was conducted in the absence of any commercial or financial relationships that could be construed as a potential conflict of interest.

## Abbreviations

A10: Dopaminergic cell group A10
A11: Dopaminergic cell group A11
A13: Dopaminergic cell group A13
ABA: Accessory basal amygdala
AMG: Amygdala
BG: Basal ganglia
BLA: Basolateral amygdala complex
CeA: Central nucleus of the amygdala
CeM: Medial central nucleus of the amygdala
ChR2: Channelrhodopsin 2
Chx10: Transcription factor Chx10
CnF: Cuneiform nucleus
D1: Dopamine receptor subtype 1
D2: Dopamine receptor subtype 2
DDS: Diencephalospinal dopamine system (in zebrafish)
dlPAG: Dorsolateral periaqueductal gray
DLR: Diencephalic locomotor region
dMSNs: Direct pathway medium spiny neurons
dPAG: Dorsal periaqueductal gray
GABA: gamma-Aminobutyric acid
GAD65: glutamic acid decarboxylase
Gi: Gigantocellular reticular nucleus
GiA: Gigantocellular reticular nucleus alpha part
GiV: Gigantocellular reticular nucleus ventral part
GP: Globus pallidus
GPe: Globus pallidus external
GPi: Globus pallidus internal
iMSNs: Indirect pathway medium spiny neurons
LH: Lateral hypothalamus
Lhx3: Transcription factor Lhx3
lPAG: Lateral periaqueductal gray
LPGi: Lateral paragigantocellular nucleus
MdV: Ventral part of medullary reticular formation
MLR: Mesencephalic locomotor region
MRF: Medullary reticular formation
NAc: Nucleus accumbens
PAG: Periaqueductal gray
PMH: Premammillary nucleus of the hypothalamus
PnC: Pontine reticular nucleus caudal
PnO: Pontine reticular nucleus oral
PPN: Pedunculopontine nuclei
PPTg: Pedunculopontine tegmental nucleus
SC: Superior colliculus
SLR: Subthalamic locomotor region
SN: Substantia nigra
SNc: Substantia nigra pars compacta
SNr: Substantia nigra pars reticulata
STh/ STN: Subthalamic nucleus
V1: Primary visual cortex
V1 L5: Layer 5 of the primary visual cortex
Vglut2: Vesicular glutamate 2
vlPAG: Ventrolateral periaqueductal gray
VMH: Ventromedial hypothalamus
VP: Ventral pallidum
VTA: Ventral tegmental area
ZI: Zona incerta.
